# The Platinum Rule: A New Standard for Person-Centered Care

**DOI:** 10.1089/jpm.2022.0075

**Published:** 2022-05-18

**Authors:** Harvey Max Chochinov

**Affiliations:** ^1^ Department of Psychiatry, University of Manitoba, Winnipeg, Manitoba, Canada.; ^2^ CancerCare Manitoba Research Institute, Winnipeg, Manitoba, Canada.

**Keywords:** bias, distorted compassion, patient autonomy, advance care planning, patient values, therapeutic nihilism

## Abstract

How decisions are made and patients cared for are often guided by the Golden Rule, which would have us treat patients as we would want to be treated in similar circumstances. But when patients' lived experiences and outlooks deviate substantively from our own, we stop being a reliable barometer of their needs, values, and goals. Inaccurate perceptions of their suffering and our personal biases may lead to distorted compassion, marked by an attitude of pity and therapeutic nihilism. In those instances, The Platinum Rule, which would have us consider *doing unto patients as they would want done unto themselves,* may be a more appropriate standard for achieving optimal person-centered care. This means knowing who patients are as persons, hence guiding treatment decisions and shaping a tone of care based on compassion and respect.

Bert was a kind 74-year-old happily married gentleman and father of five children. He had smoked cigarettes for a few decades, but had quit years ago, yet had presented with a cancer in his mouth. He underwent a large surgery that left him hoarse and disfigured. He was unable to swallow and depended on a gastrostomy tube for his feedings. Chemotherapy and radiation took their turns in causing more difficulties with nausea and some painful radiation effects.

Eventually the cancer recurred. More chemotherapy did not affect the tumor, and radiation was given with palliative intent. He began to have more pain, and at that point, one of his oncologists sat down with him and his wife and told them that he likely had little time to live, that his tumor was most likely going to progress quickly, and that his last days would become much more difficult, with increasing pain. The oncologist suggested that he might consider Medical Assistance in Dying (MAiD), to avoid what was sure to be a time of significant suffering.

Bert and his wife were a religious couple who had relied on prayer and the community around them to get them through over the years. They could not agree to MAiD. It was just not on their list of potential options. When he met with the palliative care consultant, he was having increasing pain, which was felt to have a large neuropathic component. A mix of gabapentin and small doses of methadone helped to reduce his pain to a very manageable level. The addition of immunotherapy by another oncologist resulted in a surprisingly good outcome, and now six months later, although still depending on gastrostomy feedings, he is frequently out in the garden, watering and weeding, and hoping to take part in harvest. He recently indicated his quality of life was excellent (C. Woelk, pers. comm.).

The Golden Rule—*do unto others as you would have them do unto you*—conveys deep wisdom, which can be found in some form in many religious and ethical traditions. In medicine this means treating patients and families the way we would want to be treated or would want our loved ones to be treated in similar circumstances. The Golden Rule is based on the idea of reciprocity and being able to see ourselves in others. *If I were that patient, how would I want to be treated? What if this was my spouse, my child, my parent or sibling, how would I want them to be treated?* In most instances adherence to The Golden Rule leads to health care decisions and clinical attitudes that are compassionate and embrace the essence of person-centered care.

The Golden Rule, however, has its limitations, as it requires some overlap between how we see ourselves and how others see themselves. So long as the patient's values and priorities align with our own, we can infer their needs based on how we would want to be treated in their situation. The more our worldview and lived experience deviates from theirs, the more the Golden Rule begins to unravel. *How would I want to be treated it I were that old? If I were that dependent? Or that disabled, disfigured, marginalized, or disease ridden?* Our own biases and perceptions of current, and the possibility of future, suffering can lead to attitudes that are tone deaf and decisions that are discordant with patients' perceptions, values, and goals.

What happens when, from an alleged vantage point of beneficence, we perceive someone to be suffering, based on how we imagine we would suffer in their situation? Unconscious bias can influence the way we process patient information, affecting our behavior, interactions, and decision making.^1^ A sense of therapeutic nihilism and clinical passivity can set in, a feeling that nothing is worth trying and certain lives may not be worth preserving, leading us to withhold treatment, perhaps forgo diagnostic tests and *let nature take its course*. Inferring we would not want to live this way, distorted compassion—that is compassion based on tainted or inaccurate perceptions of another person's suffering—can lead to ostensibly well-intended advice, actions, or inactions that may be completely at odds with what the patient really wants. Rather than feeling that they have been heard, distorted compassion can result in patients feeling devalued, misunderstood, and further demoralized at the very hands of those who are meant to help.

Catherine Frazee, a pre-eminent disability rights advocate, who lives with spinal muscular atrophy says, “having to wear diapers and drooling are highly stigmatized departures from what is expected of adult bodies. Those of us who deviate from these norms experience social shame and stigma that erodes resilience and increases vulnerably. The more deeply these stigmatized accounts are embedded in our discourse and social policy, the more deeply virulent social prejudice takes hold within our culture. [] What assurance can we offer that the physician who treats these adults at end-of-life will not stand at their bedside with horror or revulsion in his heart?”^2^ Adhering to the Golden Rule, we may find ourselves responding with pity and implicit or explicit encouragement for patients to *let go*, despite their determination to *hang on*.

The *Platinum Rule,* which would have us consider—*doing unto patients as they would want done unto themselves—*offers a standard that is more likely to result in treatment decisions that are consistent with patients' personal needs and objectives. *Doing unto* as per the Platinum Rule implicates not only clinical decisions, but treating patients—as in acting toward them—as they would want to be treated. This means establishing a care tenor that is informed by asking what we need to know about them as a person to take the best care of them possible.^3^

This kind of sensitivity to personhood increases the likelihood that our responses are personalized and genuinely compassionate. And when stated preferences are less certain, it is important to explore their and their family's values to inform treatment recommendations. Deep inquiry is needed from a position of cultural humility, which emphasizes “that [healthcare providers] must acknowledge the experiential lens through which they view the world and that their view is not nearly as extensive, open, or dynamic as they might perceive.”^1^ This approach requires the development of self-awareness as a critical step in achieving mindfulness for others.^4^

Of course, not all patient preferences can or should be accommodated, especially when they are driven by nihilistic self-loathing (*I don't want anything*), or motivated by expectations that exceed any objective reality (*I want everything*). Even then, it is important to understand their wishes, and what approaches might provide them with optimal comfort and reassurance. Although this may see attitudes and therapeutic considerations shift away from our own reflexive inclinations, a platinum standard acknowledges that we cannot always be the perfect infallible barometer of our patients' preferences, values, and goals.

The Platinum Rule also applies when guiding substitute decision makers. The question they must consider is not what they would want done, but what the patient would want done in this instance. *Imagine your critically ill dad six months ago and tell me what he would want us to do. Let's sit at his bedside and imagine saying ‘okay dad, you've been in hospital for two weeks. You've been unconscious for two days. The doctor says he doesn't think you are going to make it through the night, but he also thinks you have pneumonia, which in theory is treatable, but nobody knows how you're going to respond to that* (M. Harlos, pers. comm.).

The question is not what the substitute decision maker would want done, but what the father would want done *unto himself*—The Platinum Rule. This aligns with a substitute judgment standard,^5^ wherein surrogates are asked to make decisions that patients would have made if they were competent. However, the Platinum Rule goes beyond simply trying to intuit what patients might want when they are unable to voice their preferences and implicates being able to tap into exquisite sensitivity for how all patients would wish to be perceived and treated. This requires confronting personal biases that might cause us to respond to patients according to our own ingrained perceptions and values—defaulting to a Golden standard—when nothing less than a Platinum standard will do.

Giving him the benefit of the doubt, one can easily imagine Bert's physician recommending MAiD from a position of wanting to mitigate current and future suffering. One can also easily imagine, based on the Golden Rule, that he offered a solution for a clinical situation he could neither fathom himself nor those he loved being able to bear. Distorted compassion, however, represents a failure of the imagination. Perceptions of suffering can obstruct our ability to imagine patients experiencing life as having sustained meaning, purpose, and value, despite even overwhelming challenges. The Golden Rule has its place in medicine, given it provides an initial gauge in our response to patient suffering. But if we are truly intent on offering patient-centered care, consistent with *their* values, preferences, and goals, consideration of the Platinum Rule is required: *doing unto patients as they would want done unto themselves.*
